# Laparoscopic Groin Hernia Repair Using the Totally Extraperitoneal Approach: A Retrospective Study and Our Experience

**DOI:** 10.7759/cureus.41151

**Published:** 2023-06-29

**Authors:** Rafique Umer Harvitkar, Giri Babu Gattupalli, Hashim Al-Hano, Khaled F Al-Kharouf, Abhijit Joshi

**Affiliations:** 1 General Surgery, Queen Alexandra Hospital, Portsmouth, GBR; 2 Surgery, Sri Chandra Sekara Hospital, Hosur, IND; 3 Surgery, Queen Alexandra Hospital, Portsmouth, GBR; 4 Surgery, Dr L H Hiranandani Hospital, Mumbai, IND

**Keywords:** occult hernia, seroma, turp, inguinal hernia, tep repair

## Abstract

Background

Recently, laparoscopic totally extraperitoneal (TEP) inguinal hernia repair has been considered one of the most effective and widely performed techniques for repairing inguinal hernias by avoiding entry into the peritoneal cavity. Its indications have evolved and expanded to almost encompass the entire range of groin hernias. This retrospective study aims to determine the outcomes and postoperative complications in patients undergoing TEP inguinal hernia repair performed by a single surgeon for groin hernias at a single center.

Methodology

We retrospectively evaluated the prospectively collected data of 900 patients who underwent elective TEP repair over 18 years at a single center performed by a single surgeon from April 2004 to February 2023. Patients were evaluated for age, sex, type of hernia, time taken for surgery, open from laparoscopy, intra and postoperative complications, hospital stay, and days taken to resume regular activity.

Results

The mean age of the 900 patients was 59 years (range = 21-83 years). The mean age of males and females was 59 and 56 years, respectively. The mean operative time was 40 and 55 minutes for a unilateral and bilateral hernia, respectively. In total, 369 (41%) patients had a right-sided groin hernia, 382 (42%) patients had a left-sided groin hernia, and 149 (16.5%) patients had bilateral groin hernias. A total of 121 (13%) patients had occult hernias, and 17 patients underwent concurrent TEP and transurethral resection of the prostate. Of the 900 patients, 20 (2.2%) had a recurrent hernia after a previous open inguinal hernia repair. Seven (0.8%) patients had a recurrence of hernias post-TEP and subsequently underwent open inguinal hernia repair. Seven (0.7%) patients needed conversion from TEP to the transabdominal pre-peritoneal approach. Only minor complications were noted intra and postoperatively. The average time of hospitalization was 24 hours. The time to resume normal activities was five (±1) days.

Conclusions

Our experience suggests that TEP repair with mesh fixation is a safe and effective procedure with a marginal recurrence rate. Apart from the obvious cosmetic benefits of minimal tissue invasion, a significant advantage of TEP is the visualization of the contralateral groin along with the surgical repair of a hernia, if required, in the same sitting and without the insertion of any extra trocars.

## Introduction

The first laparoscopic approach for inguinal hernia repair was reported by Ger in the early 1990s [1]. Over time, various transabdominal laparoscopic techniques evolved, with transabdominal, preperitoneal, and intraperitoneal-only procedures being the most common ones. The totally extraperitoneal (TEP) technique was first described by Ferzli (1992) and McKernan (1993) with outcomes compared to available methods. The TEP approach was developed because of concerns for possible complications related to intra-abdominal procedures, which included transabdominal preperitoneal (TAPP) and other methods [2-4]. The ideal procedure for the surgical repair of an inguinal hernia has been a subject of controversy and debate in the surgical fraternity since its inception. The history of inguinal hernia repair over the years and decades reveals how metamorphosis is incorporated into surgical practice via the amalgamation of subjective and scientific processes. The development of laparoscopic procedures for inguinal hernia repair aligned with modern-day technologies and individual experiences. The laparoscopic approach was adopted as a tension-free approach well documented by Nyhus and Stoppa in their open techniques for hernia repair.

Inguinal hernia is a well-known entity worldwide, with a prevalence of 1.6% in adults, which increases to 4.3% in patients over 46 years of age, accounting for 75% of all abdominal wall hernias. Its incidence increases with age. Inguinal hernia affects the male population more commonly, with a risk of 26% versus 4% noted in females [5-7]. Several studies have illustrated the benefits of minimally invasive hernia repair over conventional open repair, including less postoperative pain and early recovery [7]. TEP and TAPP are the most widely accepted approaches for inguinal hernia repair. There are several fundamental differences between the two approaches. The TEP approach circumvents abdominal cavity entry and avoids the issue of peritoneal closure. It can be performed with minimal use of electrocautery, thereby enabling less postoperative pain. This article describes our experience with TEP performed by a single surgeon over 18 years at a single center.

## Materials and methods

A retrospective study of elective laparoscopic TEP inguinal hernia repair was conducted between April 2004 and February 2023 at a tertiary care hospital. All hernia repairs were performed by a single surgeon. The data were collected from the hospital’s electronic medical records (EMRs). A total of 900 patients were included in this study. The diagnosis of inguinal hernia was based on medical history, clinical examination, and radiological investigation (mainly an ultrasound scan). Once diagnosed, patients were advised to undergo a pre-anesthesia check-up and routine investigations to assess their fitness for surgery and anesthesia. Males over 40 years of age with relevant symptoms were evaluated for prostatomegaly and, if required, a consultation was arranged with a specialist consultant in urology. After confirming surgical fitness, patients were advised to come to the hospital three hours before surgery on an empty stomach after a nighttime meal. Surgery was performed on the day of the admission to the hospital. Only those who fulfilled the inclusion criteria were subjected to TEP repair. Our inclusion criteria were all patients with uncomplicated groin hernias, unilateral or bilateral hernias, primary or recurrent post-open rehabilitation, and the American Society of Anesthesiologists up to grade 3. Our exclusion criteria were all patients who had undergone previous preperitoneal surgery (hernia, prostate, vascular, kidney transplant); those with ascites, strangulated hernias, and giant scrotal hernias; septic patients; those with a previous laparotomy with am infra-umbilically extended incision; those with severe cardiovascular and respiratory compromise; those with the hemostatic disorder; and those with hemodynamic instability or hypercapnia of more than 50 torrs.

All surgeries were performed on a one-night-stay basis, with discharge on the next day. Patients were advised to take analgesics (paracetamol for patients with compromised renal function and ibuprofen + paracetamol for others) as and when required, as well as to avoid strenuous physical activity for the first eight weeks after the surgery. All surgeries were done by the same surgeon under general anesthesia. Data for the following parameters were obtained from the EMRs: age, sex, type of hernia, duration of surgery, conversion from TEP to TAPP, intra and postoperative complications, duration of hospital stay, and time to resume regular activity.

Follow-up was done on postoperative day 10 in the outpatient department (OPD) and then telephonically at three months and six months post-surgery. Patients were questioned about pain and recurrent swelling. Those who answered in the affirmative were called to the OPD for a physical examination.

## Results

This retrospective study was conducted at a single center among a total of 900 patients who were operated upon by the same surgeon between April 2004 and February 2023. There were 640 (71%) males and 260 (29%) females. The median age of the patients was 59 years (range = 21-83 years). The mean age for males and females was 59 and 56 years, respectively. A right-sided groin hernia was noted in 369 (41%) patients, 382 (42%) patients had a left-sided groin hernia, and 149 (16.5%) patients had bilateral groin hernias. A total of seven (0.77%) patients had a recurrence of hernia after TEP. Two of the seven patients underwent a right TEP, three underwent a left TEP, and two underwent a bilateral TEP. These recurrences were observed at three months (four patients) and six months (three patients) post-surgery. Subsequently, all these patients underwent Lichtenstein’s repair (open mentoplasty). The mean operative time was 40 minutes for unilateral and 55 minutes for bilateral hernia repairs.

Of the 900 patients, 120 (13%) had a concurrent occult hernia (OH). Of the 120 OH patients, 116 (96.7%) were males, and four (3.3%) were females. However, 115 (95%) patients had unilateral OH (UOH), and five (4.5%) patients had bilateral direct OH (BOH). Of these 115 patients, 70 (61%) patients with UOH had a right inguinal hernia, whereas 40 (34%) patients had a left inguinal hernia. Two patients had a right femoral and pantaloon hernia each (0.86%). However, one (0.86%) patient had a right Spigelian hernia. Of these four females, two (50%) had UOH, whereas the remaining two had BOH. One UOH had an ipsilateral direct inguinal hernia, while the other had an ipsilateral femoral hernia.

The two female patients with BOH had bilateral direct inguinal OH and bilateral femoral OH, respectively. Of the 120 patients with OH, 117 (97.5%) had UOH, while three (2.5%) had BOH. Among the three patients with BOH, one was male (bilateral direct OH), and two were female (Table 1).

**Table 1:** Patients demographics. TEP: totally extraperitoneal; TURP: transurethral resection of the prostate

Patients characteristics	N (%)
Total number of patients	900
Male/Female	640/260 (71%/29%)
Age (range)	21–83 years
Hernia side	
Right groin	369 (41%)
Left groin	382 (42%)
Bilateral	149 (16.5%)
Operating time	
Unilateral hernia	40 minutes
Bilateral hernia	55 minutes
Occult hernia	120
Unilateral inguinal	115 (95%)
Bilateral inguinal	5 (5%)
Femoral hernia	2 (1.8%)
Spigelian hernia	1 (0.8%)
TEP + TURP	17 (1.8%)

In our series, out of the 900 patients operated on with TEP, 17 also required a concurrent transurethral resection of the prostate (TURP), as decided by our specialist urological surgeon during his preoperative consultation. Breaking with the established convention, we performed concurrent TEP and TURP on these 17 patients and published our results. In the entire series, there was no conversion to open repair. However, we converted seven (0.77%) patients to TAPP due to technical difficulties. Two patients had significant intraoperative bleeding from the inferior epigastric vessel during trocar insertion. One patient was managed with bimanual tamponade after cantilevering the trocar against external pressure provided by the surgeon’s hand. In the other patient, the bleeding was controlled by cauterizing the inferior epigastric vessels using bipolar coagulation. Only minor complications were noted postoperatively. Patients were followed up on postoperative day (POD) 10 with a physical follow-up and telephonically at three months and six months post-surgery as part of the study protocol. In total, 35 (3.8%) patients developed postoperative seroma at the first follow-up on POD 10. Only three remained at eight weeks and none at 12 weeks. All these were typically patients with large, complete, indirect sacs where absolute intraoperative sac reduction was impossible. Two patients required a single sitting of ultrasound-guided aspiration of seroma performed at two weeks and five weeks post-surgery, while the remaining patients improved conservatively. Each case of seroma was confirmed with an ultrasound scan for recurrence of the hernia.

A total of four (0.5%) patients had a scrotal hematoma noticed 48 hours post-surgery, which resolved within two weeks of surgery after conservative management. There was no immediate or delayed postoperative bleeding from the surgical sites. None of the patients, including the 17 who underwent concurrent TEP and TURP, had mesh infections. Ten (1.25%) patients required postoperative catheter insertion for urinary retention. All 10 patients were males over the age of 50 years. Their admission was continued for another 24 hours, and they were discharged on POD one after a successful voiding trial. The median pain score at 12 weeks post-surgery was zero on the Visual Analog Scale (VAS). There was a complete resolution of preoperative symptoms in all patients. However, 12 (1.3%) patients described intermittent discomfort in the groin only during extreme physical effort, which was of little concern to them and required no medication. The average VAS score of these 12 patients was 1. None of them had a recurrence of the hernia. The average time to resume normal activities was five days post-surgery.

Procedure

All procedures were performed in a supine position under general anesthesia. The semi-lithotomy position was utilized for TURP and the prone position for the TEP procedure. Seven patients underwent TEP followed by TURP, whereas 10 patients underwent TURP followed TEP. Third-generation intravenous cephalosporin was the antibiotic of choice for each patient per trust guidelines. Optimum triangulation using the three-trocar technique was adopted for the TEP repair. Preperitoneal entry was gained via a sub-umbilical incision using a 10 mm optical trocar.

**Figure 1:**
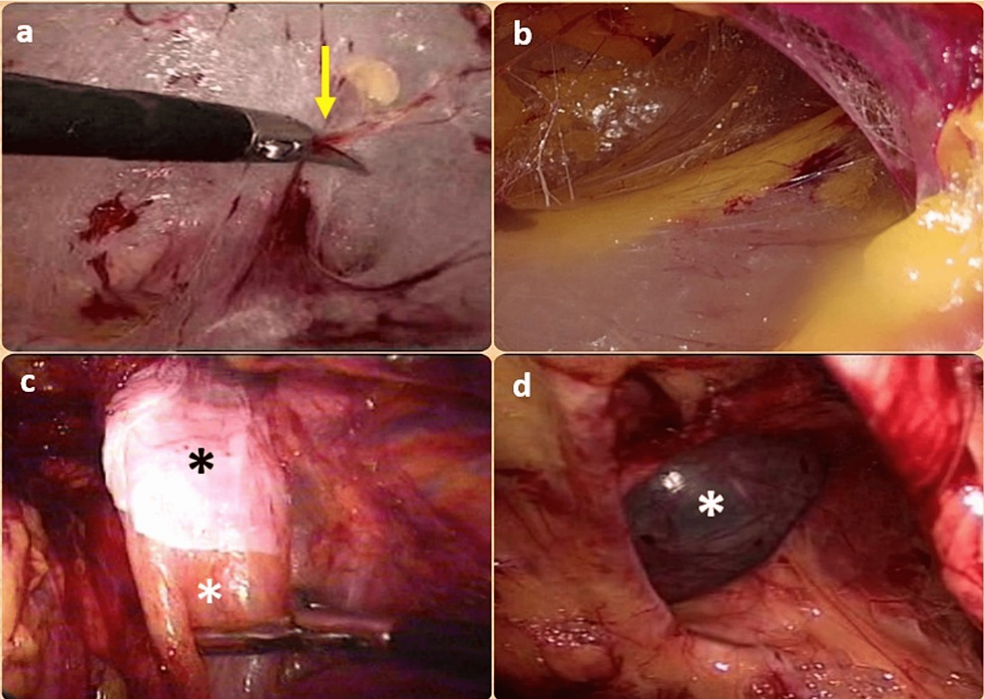
(a) Development of the preperitoneal space with an initial blunt telescopic and then sharp dissection (yellow arrow). (b) Developed preperitoneal space. (c) Separation of the true sac (white asterisk) from the pseudo-sac (black asterisk). (d) Bare direct hernial defect (white asterisk) after complete reduction of the sac.

Blunt telescopic dissection was performed under direct vision using a zero-degree scope to develop preperitoneal space (Figure 1, Panel a). Post-development of space (Figure 1, Panel b), the telescope was switched to the 30 degrees one, and the rest of the ports (5 mm) were inserted under direct vision.

Contralateral trocars were inserted first for a unilateral hernia to facilitate dissection in the ipsilateral space. The spino-umbilical line is used as a marker to insert the working trocars. The contralateral trocar was inserted 2-3 cm below the mid-point of this line. In contrast, the ipsilateral trocar was inserted on the mid-point of the ipsilateral line to prevent the instruments from “fighting” with each other and the telescope. Attempts were always made to separate the true sac from the pseudo-sac (Figure 1, Panel c).

**Figure 2:**
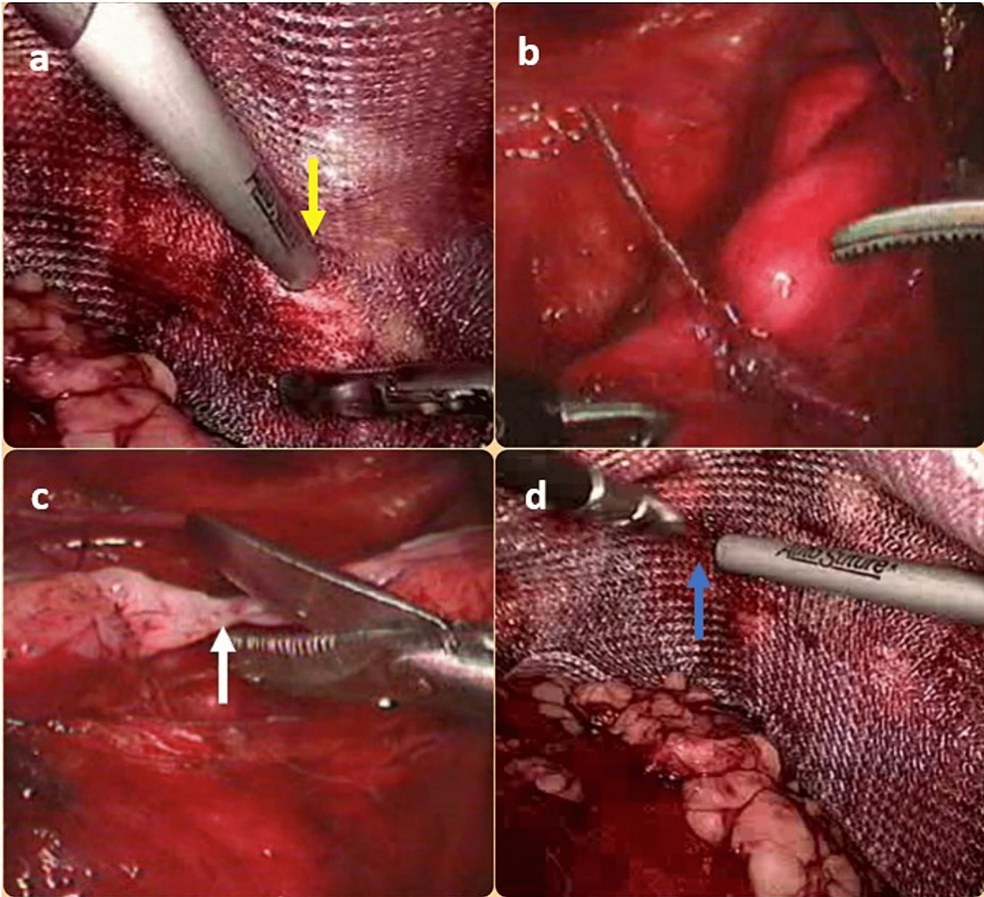
(a) Tack fixation of Prolene mesh to Cooper’s ligament (yellow arrow). (b) Ligated and skeletonized indirect inguinal hernia sac. (c) Transection (white arrow) of the ligated indirect sac. (d) Tack fixation of Prolene mesh to parietes (blue arrow).

In a direct hernia, the sac was permanently reduced completely (Figure 1, Panel d). However, in indirect sacs, gentle attempts were made to minimize the sac. It is sometimes tricky to ultimately reduce chronic long-standing sacs. In some of these cases, the sac was skeletonized, ligated, and transacted (Figure 2, Panels a-c).

In cases of bilateral hernias, the first 5 mm trocar was always inserted on the side of the minor hernia, followed by the rest of the steps mentioned above.

**Figure 3:**
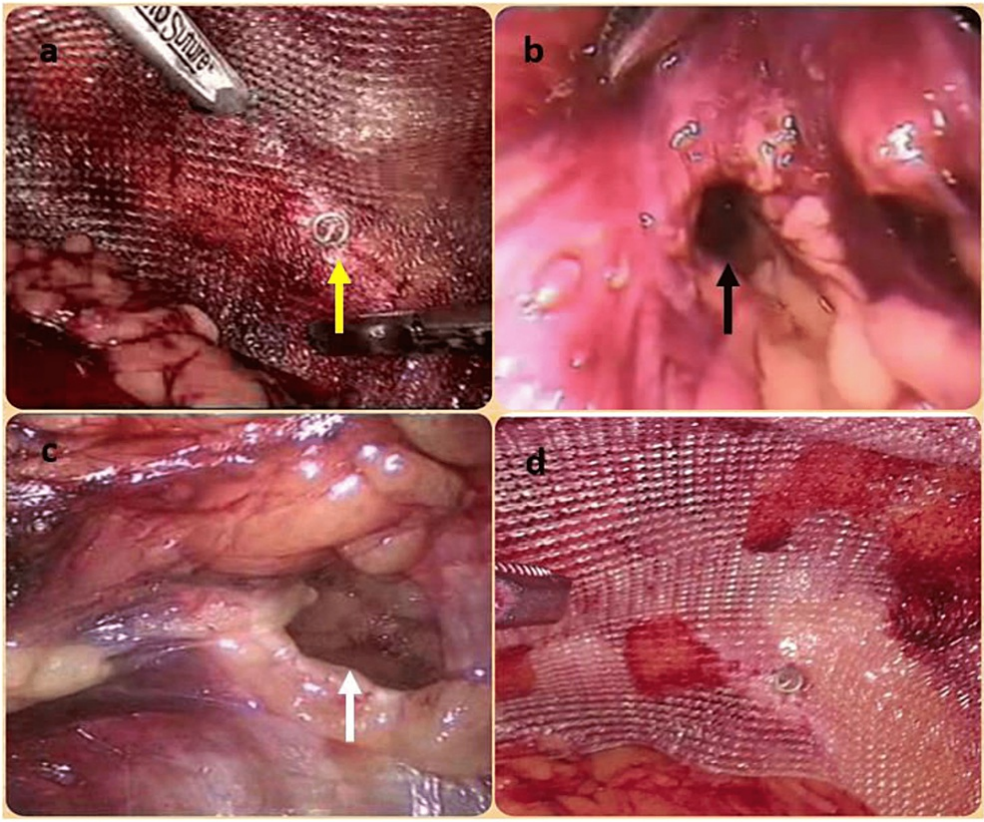
(a) Titanium tack (yellow arrow) fired through the mesh into Cooper’s ligament, thereby fixing the mesh. (b) Occult femoral hernia (black arrow) incidentally found during repair of the ipsilateral indirect inguinal hernia. (c) Occult Spigelian hernia (white arrow) incidentally found during repair of the ipsilateral direct inguinal hernia. (d) Prolene mesh optimally placed and tack-fixed to cover all potential groin hernial orifices.

Careful dissection was performed in and around the vas deference avoiding excessive handling. Adequate lateral, medial, and proximal space was created to accommodate the mesh. A polypropylene mesh (weight: 75 g/m^2^, pore size: 0.75 mm, size: 15 × 11 cm) was used as a prosthesis. This was fixed to Cooper’s ligament and abdominal wall with the help of tacks and to the parties (Figure 2, Panel d; Figure 3, Panel a). In cases of bilateral hernias, two meshes were used with central overlapping.

To minimize contamination, two separate trolleys were used in patients undergoing concurrent TEP and TURP.

During ipsilateral dissection, with awareness and a little extra effort, we can visualize all ipsilateral groin hernial orifices other than the site of the clinically discerned and diagnosed hernia.

These are the sites for occult ipsilateral hernias. Careful attention was drawn to the femoral and obturator orifices after dealing with a clinical hernia sac to look out for minor defects or indentations. The lateral rectus abdominis was visualized to look for a Spigelian hernia (Figure 3, Panels b,c).

Per departmental policy and protocol, once we finished with the clinical hernia, the contralateral side was dissected to look for occult hernia irrespective of clinical suspicion. Once found occult on the contralateral side, an appropriately sized mesh was covered over the clinical and occult defect.

If a single large mesh did not cover both defects, an extra small mesh was overlapped with the larger mesh (Figure, Panel d). This is usually true for Spigelian or obturator hernias, where the defects are more laterally spread. In occult femoral hernias, the fault usually gets covered with one large mesh by pulling the mesh a little more caudally (Table 2).

**Table 2:** TEP steps. TEP: totally extraperitoneal

TEP steps
Identification of the symphysis pubis in the midline
Opening the space of Retzius by a blunt and sharp dissection along Cooper’s ligaments
Hesselbach’s triangle identification with potential related hernias
Identification of epigastric vessels
Development of the space of Bogros up to the level of the anterior superior iliac spine
Identification and separation of cord structures and sac reduction
Placement of a mesh covering all defects

## Discussion

TEP repair for inguinal hernia is a globally accepted procedure with a low recurrence rate in experienced hands. However, the optimum procedure for inguinal hernias remains debatable [8]. Per the National Institute for Health and Care Excellence, both open and laparoscopic surgeries are considered safe and recommended procedures. They emphasize that individuals should be fully informed about the pros and cons of the surgery. Key decision-making factors are the patient’s fitness for general anesthesia; whether the patient has a primary, recurrent, unilateral, or bilateral hernia; and the surgeon’s experience in performing the procedure.

Minimally invasive TEP repair offers clear benefits, such as less postoperative pain, early recovery, and a decreased incidence of chronic pain and numbness. TEP is the preferred technique for recurrent hernias, as scar tissue from previous open repairs can be avoided. Although TEP is considered a technically more challenging surgery than TAP, it has a low risk of damaging intra-abdominal organs and a lower chance of forming adhesions. According to statistics, approximately 21 million cases of inguinal hernias are treated surgically every year [9]. Among the surgical methods used for hernia repair, the most common techniques are TEP and TAP [10]. According to previously published data, the TEP procedure has a shorter time than TAP. In addition, TEP has a high threshold for postoperative pain and a lower postoperative analgesic requirement. This may be due to the short duration of surgery, the intra-abdominal approach, and the lack of sutures on the peritoneum [11]. TEP facilitates the complete separation of the preperitoneal space without entering the peritoneal cavity. This procedure has a restricted operative field which increases the difficulty level of the procedure, especially for inexperienced surgeons. It also leads to a steep and long learning curve [4,12]. Successful insufflation of the preperitoneal space is a crucial factor for ensuring the success of the procedure, especially when entering the space of Bogros from the Retzius area. If the peritoneum is breached during this procedure, gas enters the peritoneal cavity, thereby bulging the peritoneum into the operative field and further narrowing the field of vision and surgery. This is a common reason for the failure of TEP. However, in such cases, inserting a Veress needle at Palmer’s point helps in deflating the peritoneum and facilitating the remainder of the surgery.

Fixation of mesh has not been advocated by many surgeons due to concerns about constant pain and tenderness over the fixation site. However, in patients with significant hernial defects, the mesh can be fixed to three points, namely, the symphysis pubis, Cooper’s ligament, and the anterior abdominal wall (lateral to inferior epigastric vessels). Mesh fixation in the preperitoneal space is a widely debated issue. Numerous methods have been suggested and described. The placement of sutures laparoscopically is technically more challenging and time-consuming in such a small space. In our institute, we use titanium tacks (Protack®, Covidien Inc.) for mesh fixation. A recent study advocated the use of biodegradable adhesive for mesh fixation [2,11-13]. This adhesive keeps the mesh in place until fibrosis occurs. Post-fibrosis, the glue degrades and gets absorbed. This method circumvents the need for tacker firing for mesh fixation. Although this is a newer technique with limited fixation, it requires further evaluation and research to validate the technique. Overall, the morbidity with TEP repair is 4-8% of patients experiencing minor complications such as wound infection and bruising, which are managed conservatively in most instances [14]. However, surgeons should be aware of serious complications such as bladder injury, injury to the vas, and vascular injury, and their incidences are not more than 0.6% [14-18].

## Conclusions

The laparoscopic TEP procedure has been well-established as the preferred surgical therapy for uncomplicated groin hernias. As seen in this paper about our experience with TEP, an additional benefit is the visualization of the contralateral groin through the same three ports. This enables the diagnosis of a possible concurrent contralateral groin hernia (occult or otherwise), logically leading to its concurrent repair in the same sitting, thereby sparing the patient another surgery later. Moreover, the small sub-series of 17 patients in the present study who underwent concurrent TEP and TURP while breaking the established convention about not performing these two operations concurrently shows that this is feasible without any risk of mesh infection. This underscores the significant advantage to the patients of getting both surgeries done under the same session of anesthesia, thereby avoiding a separate second admission and surgery. Although we have not performed any cost-benefit analysis, this single-stage approach for carefully selected patients will undeniably reduce the fiscal burden on the healthcare infrastructure. Our data regarding post-TEP hernia recurrence and post-procedure complications show that both are at par with or below the statistics in the English-language literature.
